# Normal patellofemoral kinematic patterns during daily activities in dogs

**DOI:** 10.1186/s12917-016-0889-z

**Published:** 2016-11-25

**Authors:** Erica J. Moore, Stanley E. Kim, Scott A. Banks, Antonio Pozzi, Jason D. Coggeshall, Stephen C. Jones

**Affiliations:** Comparative Orthopaedics Biomechanics Laboratory, College of Veterinary Medicine, University of Florida, PO Box 100126, 2015 SW 16th Ave, Gainesville, FL 32610-0126 USA

**Keywords:** Gait analysis, Dog, Kinematic, Patellofemoral

## Abstract

**Background:**

Patellar abnormalities are a common cause of pain and lameness in dogs; however, in vivo the relative motion between the femur and patella in dogs is not well described. The objective of this study was to define normal in vivo sagittal plane patellofemoral kinematics in three axes of motion using non-invasive methods. We hypothesized patellofemoral alignment in the sagittal plane would tightly correlate with the femorotibial flexion angle. Six healthy dogs without orthopedic disease underwent computed tomography (CT) of their hind limbs to create 3-D models of the patella and femur. Normal stifle joint motion was captured via flat-panel imaging while each dog performed a series of routine activities, including sitting, walking, and trotting. The 3-D models of the patella and femur were digitally superimposed over the radiographic images with shape-matching software and the precise movement of the patella relative to the femur was calculated.

**Results:**

As the femorotibial joint flexed, the patellofemoral joint also flexed and the patella moved caudally and distally within the femoral trochlea during each activity. Patellar flexion and distal translation during walk and sit were linearly coupled with the femorotibial flexion angle. Offset was evident while trotting, where patella poses were significantly different between early and late swing phase (*p* ≤ 0.003). Patellar flexion ranged from 51 to 6° while trotting. The largest flexion angle (92°) occurred during sit. The patella traversed the entire proximodistal length of the femoral trochlea during these daily activities.

**Conclusions:**

Using single-plane flat-panel imaging, we demonstrated normal in vivo patellofemoral kinematics is tightly coupled with femorotibial kinematics; however, trot kinematic patterns did not follow the path defined by walking and stand-to-sit motions. Our normal data can be used in future studies to help define patellofemoral joint kinematics in dogs with stifle abnormalities.

## Background

The stifle is a complex synovial joint consisting of the femorotibial and patellofemoral joints. Stifle motion occurs in three planes resulting directly from the intricate anatomical relationship between the distal femur, proximal tibia and fibula, patella, pelvic limb musculature, and the stifle joint capsule and its associated ligaments [1]. Patellofemoral abnormalities are common in dogs [2]. Patellar luxation is a highly prevalent disorder affecting the stifles of dogs and alters stifle mechanics [2]. Cranial cruciate ligament insufficiency is also common in dogs and has been shown to disrupt normal patellofemoral joint motion [3, 4].

Abnormal motion or kinematics of joints can lead to cartilage degradation, inflammation, pain, lameness, and progressive osteoarthritis [1]. The traditional method utilized for analyzing canine stifle kinematics involves attaching reflective markers to the overlying skin of tissue landmarks with movement captured by digital cameras [5–8]. These studies, while non-invasive, do not provide precise data regarding movement of the underlying bones; instead these studies only yield general information on the angles and velocities of femorotibial flexion-extension movement. Imprecise placement of the reflective markers and skin motion can introduce variability in femorotibial kinematic data during gait analysis [8]. Other methods for evaluating stifle kinematics include radiography and goniometry; however, these methods are not able to track the position of the patella during motion in dogs in vivo [9, 10].

Patellar kinematics is well described in humans and is typically referenced in relation to the femoral trochlea; of which tilt, rotation, flexion, and shift are of particular importance [11, 12]. Despite the high prevalence of patellofemoral problems in dogs, a similar description of normal patellar motion in this species has not been well described. Research evaluating the patellar kinematics following cranial cruciate ligament transection using a 2-D digital technique in a cadaveric model found patellar flexion angle was altered, suggesting abnormal patellofemoral biomechanics may play a role in the development of patellofemoral osteoarthritis in dogs with cranial cruciate insufficiency [3]. Cadaveric studies, however, have several limitations and cannot fully simulate complex in vivo stifle biomechanics.

Joint kinematics can be quantified in a precise manner in-vivo by using fluoroscopic or flat-panel methods, where the region of interest is imaged while the subject performs tasks such as walking. In-vivo femorotibial kinematics in normal and cranial-cruciate ligament deficient stifles of dogs have been characterized using continuous horizontal-beam imaging [13–15]. To the investigators’ knowledge, in vivo dynamic patellofemoral kinematics has not been studied in dogs. The objective of this study was to define the normal in vivo sagittal plane patellofemoral kinematics in dogs during daily activities using horizontal-beam flat-panel imaging. We hypothesized patellofemoral alignment in the sagittal plane (patellar flexion-extension angle, craniocaudal translation, and proximodistal translation) would tightly correlate with the femorotibial flexion angle. We also hypothesized patellar movement would be restricted to the length of the femoral trochlea.

## Methods

Six healthy Labrador Retrievers (four males, two females) were studied. All procedures were approved by the University of Florida Institution’s Animal Care and Use Committee. Dogs underwent complete physical and orthopedic examinations prior to data collection. The mean age was 4 years (range 1–7 years) and mean weight was 28 kg (range 26–32 kg). These dogs were confirmed via computed tomographic (CT) analysis to be free of pelvic limb orthopedic abnormalities.

### Data collection

Computed tomography analyses^1^ were conducted to obtain stationary images of both hind limbs from the coxofemoral to the tarsocrural joint. Transverse images were obtained with a slice thickness of 0.5 mm.

Horizontal-beam lateral projection flat-panel images^2^ of stifles were acquired while each dog walked at a velocity of 1.1 m/s (2.5 mph) and trotted at a velocity of 2 m/s (4.5 mph) on a treadmill for three separate trials with three to five strides per trial, and while the dog underwent a stand-to-sit motion for two separate trials. Images were acquired using a pulse width of 1 ms at 30 frames per second and an image area of 400 × 300 mm, producing a pixel size of 0.39 mm × 0.39 mm and image resolution of 1,024 × 1,024 pixels. Radiographic configurations supplied a 72 kV beam with a 50 mA beam current.

### Three-dimensional model creation and coordinate assignation

**Fig. 1:**
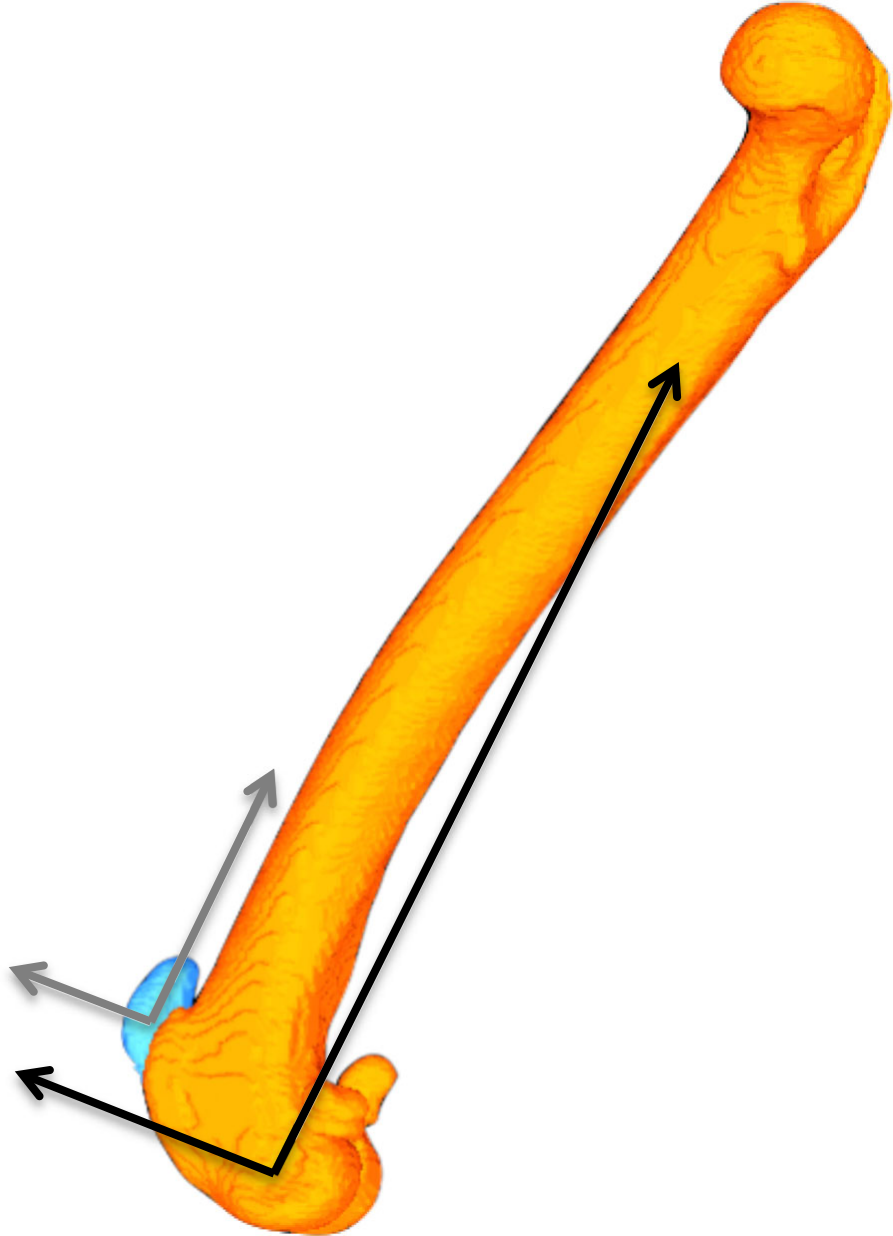
Femur and patellar coordinate systems. Patellar coordinates are indicated in gray; femoral coordinates indicated in black

Three-dimensional models of the patella and femur were constructed from the CT analyses of the subjects using the Digital Imaging and Communication in Medicine (DICOM) images and an open source 3-D-segmentation program.^3^ The bone models were imported into reverse-engineering software^4^ and coordinate systems were assigned based on local anatomical landmarks of the patella and femur [13, 16]. Femoral coordinates were applied such that the mediolateral axis (x-axis) passed through the center of the lateral and medial femoral condyles with the femoral origin located at the mid-point between the condyles (Fig. 1). The proximodistal axis (y-axis) passed proximally along the femoral shaft, perpendicular to the mediolateral axis in the plane common to the center of both femoral condyles and the femoral head. Patellar coordinates were applied such that the mediolateral axis (x-axis) passed through the most medial and lateral points on the circumference of the bone with the patellar origin defined as the mid-point of the axis. The proximodistal axis (y-axis) was defined as a line that passed through the most proximal and distal aspects of the patella, perpendicular to the mediolateral axis. The craniocaudal axes (z-axis) for the femur and patella were created from the cross product of the mediolateral and proximodistal axes, thus creating a Cartesian coordinate system.

### Three-dimensional to Two-dimensional shape-matching

**Fig. 2:**
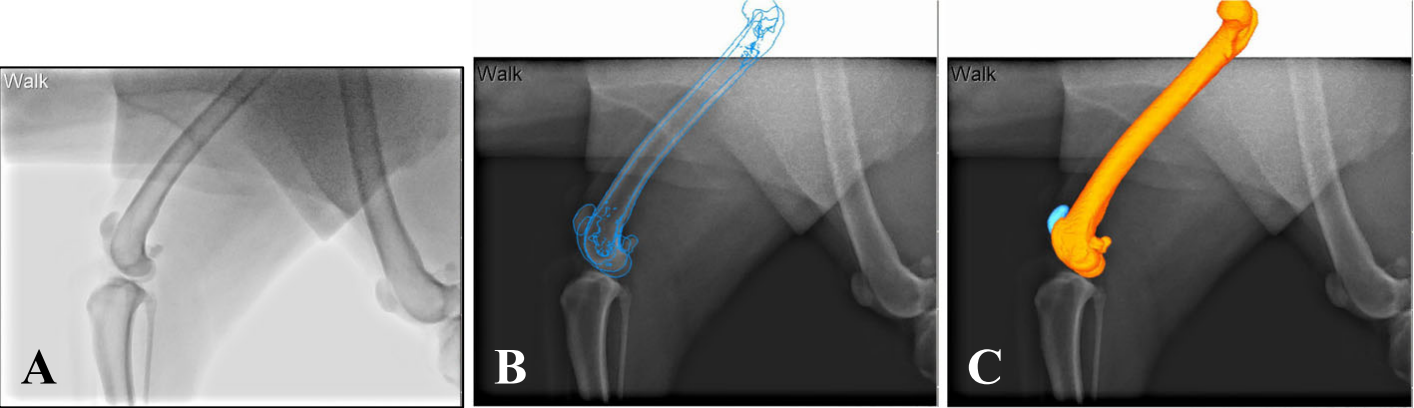
Shape-matching process. **a** Flat-panel radiographic image imported into JointTrack. **b** Contoured silhouettes based on the 3-D models of the femur and patella utilized for shape-matching over the radiographic image. **c** Shape-matched 3-D projections of the femur and patella

The 3-D bone models of the patella and femur and the single-plane flat-panel images were imported into open-source shape-matching software^5^ (JointTrack). The 3-D models were digitally superimposed over the lateral flat-panel images with JointTrack and the model’s projected silhouettes were manipulated via translation and rotation such that the anatomic contours of the models precisely overlapped the corresponding contours of the 2-D flat-panel images (Fig. 2). The patella was positioned centrally within the trochlea groove, such that the center of the patella’s articulating surface remained as congruent as possible with the trochlear groove in the axial plane. This process was performed by one individual (EM) and was repeated for each frame of the various activities and cycles performed. The orientation of the shape-matched bone models was used to calculate the relative alignment between the patella and femur for patellar flexion-extension angle (degrees), proximodistal translation (mm), and mediolateral translation (mm) using the custom-written Matlab^6^ program. The custom Matlab program transformed the data into clinically relevant patellofemoral poses in six degrees of freedom [17]. The program was utilized such that the degrees of freedom were reported as patella relative to femur. Kinematic poses were normalized and interpolated to allow for comparative analysis of each cycle shape-matched. The kinematic properties were described in relation to femorotibial flexion angle, which have been reported for this exact data set in a separate study [15].

### Statistical analysis

A statistical package^7^ was used for all analyses. Differences between single points during the gait cycle at equivalent flexion angles (offset) were determined by a paired *t*-test. A Pearson’s correlation coefficient was performed on the interpolated patellofemoral and femorotibial data to determine the extent of a linear relationship. The data was considered as strongly correlated if the r > 0.8 and weakly correlated in r < 0.3. For all statistical analyses performed, *p* < 0.05 was considered statistically significant.

## Results

**Fig. 3:**
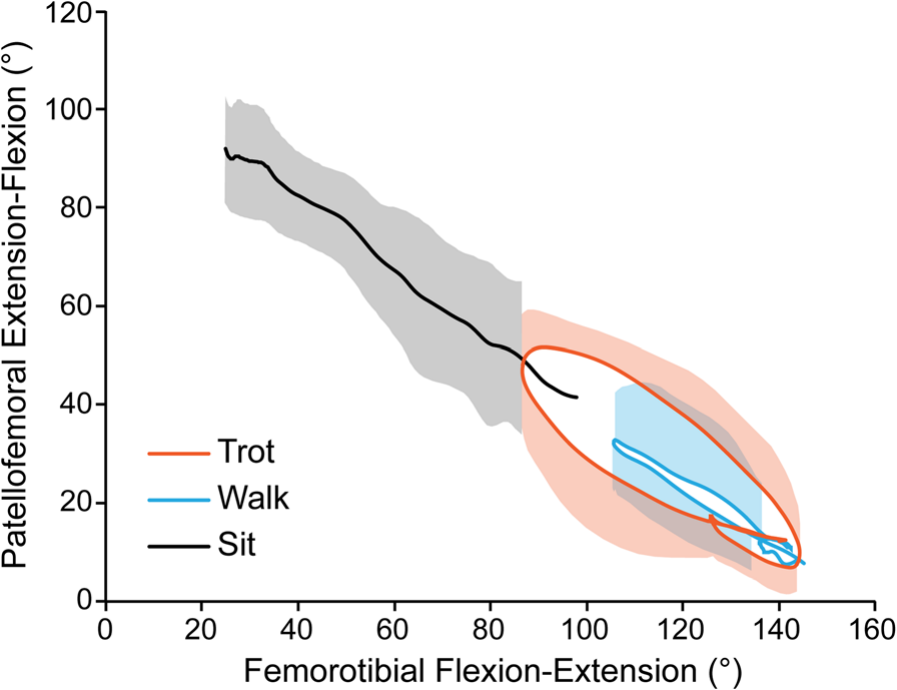
Patellofemoral flexion-extension over the course of femorotibial flexion-extension. Each color represents a daily activity of dogs and the flexion-extension patellofemoral kinematic properties are compared over the course of femorotibial flexion-extension. For the x-axis, higher values indicate greater femorotibial extension. For the y-axis, higher values indicate greater patellofemoral flexion, where the patellar long axis is less parallel to the femoral long axis

**Fig. 4:**
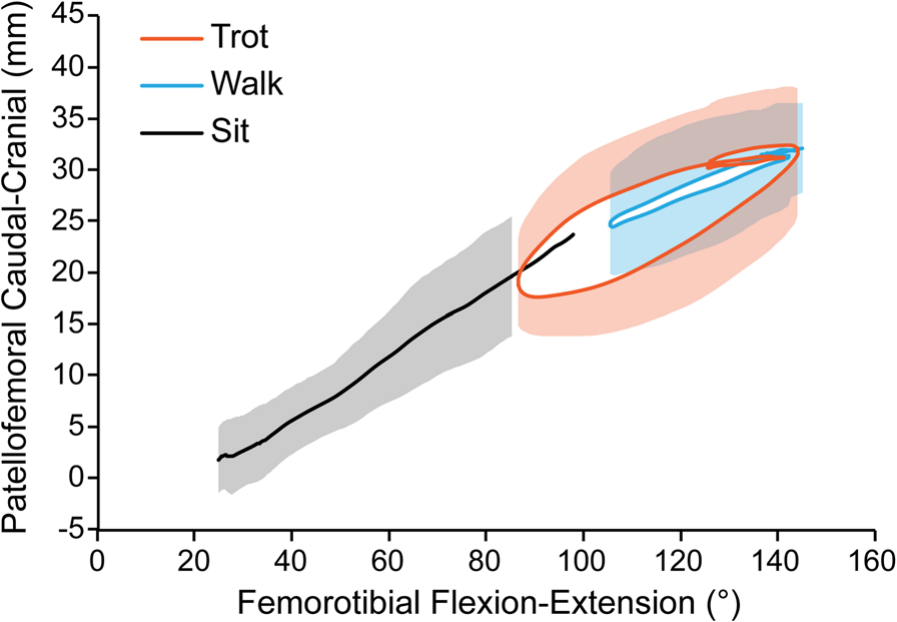
Patellofemoral craniocaudal translation over the course of femorotibial flexion-extension. Each color represents a daily activity of dogs and the craniocaudal patellofemoral kinematic properties are compared over the course of femorotibial flexion-extension. Craniocaudal translation measurements of the patella relative to the femur in mm. For the x-axis, higher values indicate greater femorotibial extension. For the y-axis, higher values indicate greater cranial translation of the patella, where the patellar origin is displaced more cranially to the femoral origin

**Fig. 5:**
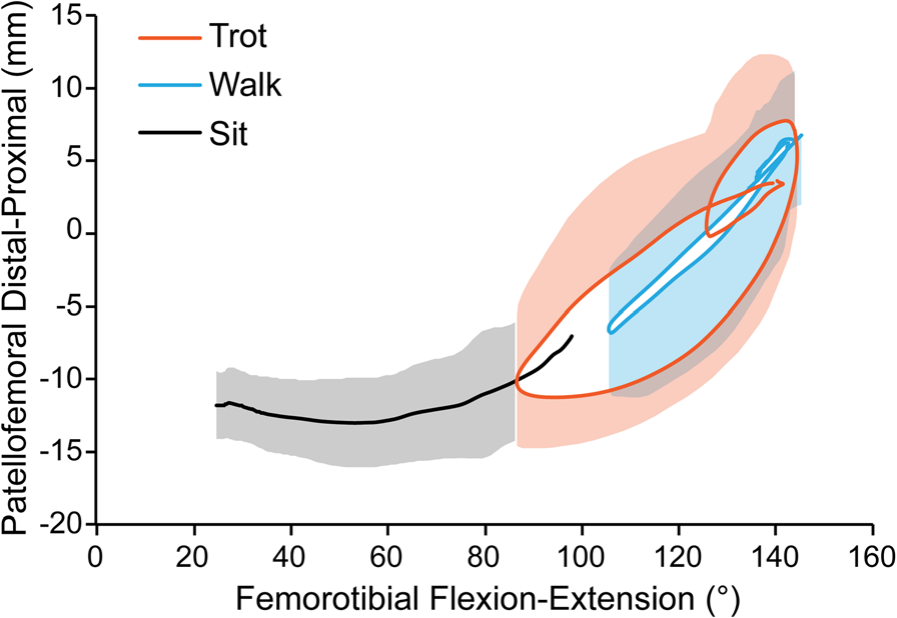
Patellofemoral proximodistal translation over the course of femorotibial flexion-extension. Each color represents a daily activity of dogs and the proximodistal patellofemoral kinematic properties are compared over the course of femorotibial flexion-extension. Proximodistal translation measurements of the patella relative to the femur in mm. For the x-axis, higher values indicate greater femorotibial extension. For the y-axis, higher values indicate greater proximal translation of the patella, where the patellar origin is displaced more proximally to the femoral origin

**Fig. 6:**
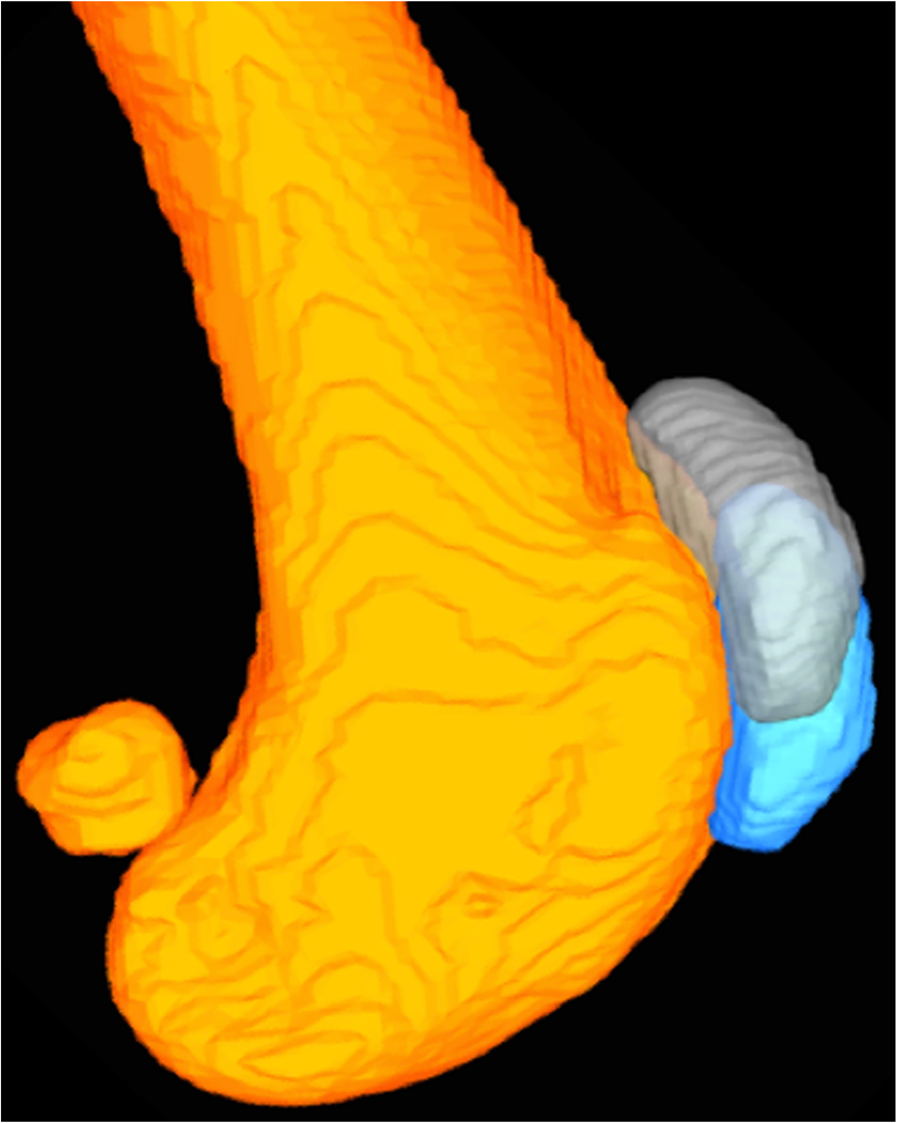
Representative image of off-set of patellofemoral poses during trot. The same femorotibial flexion angle is represented by the *orange* femur. The solid *blue* patella is the patellar orientation when entering swing phase and the *white* patella is the patellar orientation when exiting swing phase

**Fig. 7:**
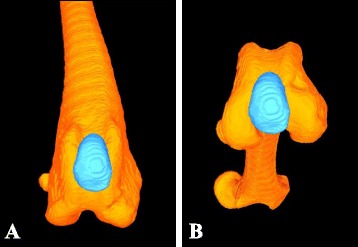
Free-view of the femur and patella during stand-to-sit motion. **a** Mean position of the femur and patella in extension at the beginning of sit. The *gray line* designates the proximal aspect of the femoral trochlea. **b** Position of the femur and patella in the flexion phase of sit with the patella appearing far distal in the femoral trochlea

As the femorotibial joint flexed, the patella also flexed (Fig. 3) and translated caudally (Fig. 4) and distally (Fig. 5) within the femoral trochlea for each activity. Patellar flexion, caudal translation, and distal translation during sit were linearly correlated with femorotibial flexion angle (Figs. 3, 4 and 5). All three measured degrees of freedom were correlated during walk and sit, however no correlation was found for these parameters during trot (Table 1). Offset, defined as significant differences in patellofemoral alignment at identical femorotibial flexion angles, was evident during trot, where the patella poses differed in early and late swing phase in each of the three measured degrees of freedom (Fig. 6; flexion-extension, *p* = 0.002; craniocaudal, *p* = 0.003; proximodistal, *p* = 0.002). Patellar flexion ranged from a mean of 51 to 6° during trot, and the largest patellar flexion angle of 92° was evident during sit (Table 1). Patellar flexion during the swing phase of walk ranged from 10° to a maximum of 33°, while a range of 7 to 13° was observed during stance (Table 2). During trot, patellar flexion angles ranged from 13 to 51° during swing and from 7 to 17° during stance (Table 2). The patella also traversed the length of the femoral trochlea during these daily activities, with nearly the entire patella positioned distal to the trochlear groove in deep flexion during sit (Fig. 7). The patella was confined within the proximal aspect of the trochlear groove during extension of all activities (Fig. 7).

**Table 1:** Average maximum, minimum, and range of motion kinematics during all three activities

	Flexion angle	Proximal translation	Cranial translation
Trot			
Maximum	51° (5)	7.8 mm (4.1)	32.4 mm (5.5)
Minimum	6° (4)	−11.3 mm (3.2)	17.6 mm (3.8)
Range of Motion	45° (6)	19.1 mm (4.1)	14.8 mm (2.7)
r	0.064	−0.029	0.008
p	0.006	0.224	0.732
Walk			
Maximum	33° (11)	6.8 mm (3.6)	32.1 mm (4.6)
Minimum	7° (4)	−6.8 mm (4.1)	24.4 mm (5.4)
Range of Motion	26° (9)	13.6 mm (2.6)	7.7 mm (3.4)
r	−0.86	0.858	0.672
p	0	0	<0.001
Sit			
Maximum	92° (12)	−13.0 mm (2.9)	23.7 mm (7.4)
Minimum	41° (19)	−7 mm (5.4)	1.7 mm (3.4)
Range of Motion	51° (20)	6.0 mm (5.1)	22.0 mm (7.5)
r	−0.951	0.346	0.893
p	0	<0.001	0

Pearson correlation coefficient (r) and p values listed in relation to femorotibial flexion angle

Data in parentheses indicate ± 1 standard deviation

**Table 2:** Average maximum and minimum during swing and stance phase during walking and trotting activities

	Flexion angle	Proximal translation	Cranial translation
Trot			
Swing Maximum	51°	3.1 mm	31 mm
Swing Minimum	13°	−11.6 mm	17.6 mm
Stance Maximum	17°	7.8 mm	32.4 mm
Stance Minimum	7°	−0.2 mm	30.2 mm
Walk			
Swing Maximum	33°	6.1 mm	31.4 mm
Swing Minimum	10°	−6.8 mm	24.4 mm
Stance Maximum	13°	6.8 mm	32.1 mm
Stance Minimum	7°	3.6 mm	31.5 mm

## Discussion

Using single-plane flat-panel imaging, we demonstrated non-invasively that normal in vivo patellofemoral kinematics are tightly coupled with femorotibial kinematics. When the daily activities were assessed collectively, we observed patellar flexion angle increased and the patella translated distally as the femorotibial joint flexed. The highest demand activity, trotting, produced kinematic patterns that did not follow the same pathways seen during walking and stand-to-sit motions. Although a portion of the patella was positioned distal to the trochlear groove during deep flexion while sitting, the base of the patella always remained positioned within the femoral trochlea during the activities analyzed in these dogs.

Our results are similar to the findings from a canine cadaveric investigation, in which passive patellofemoral joint motion was described over a smaller range of femorotibial motion [3]. Both studies demonstrated that all three kinematic parameters were linearly related to the femorotibial flexion angle. Our results, however, differ slightly with respect to the magnitude of observed change; for example, changes in femorotibial flexion angle from 90 to 150° induced proximodistal patella translation of approximately 13 mm in the cadaver study versus approximately 18 mm in our in vivo study [3]. The change in patellar flexion of approximately 25° was reported in the cadaver study, whereas we found a change of 44° over the equivalent femorotibial range of motion [3]. There are several explanations for the discrepancies between the studies. Most obviously, our investigation was an in vivo dynamic analysis, accounting for all the complex forces acting on the patella in vivo. Anatomic differences between breeds may have been a factor, as our study used Labrador Retrievers while the cadaver study used mix-breed dogs. Equally importantly, variations in coordinate assignation and reference points have been shown to dramatically affect patellar tracking patterns in humans, even within the same individual [12]. To the author’s knowledge, this is only the second report characterizing patellar kinematics in dogs. Standardized coordinate systems and reference points for future studies would allow more meaningful comparison between investigations.

The most significant finding of the study was that the relationship between patellar poses and femorotibial flexion angle varied according to the phase of the gait cycle during trotting; the patella was positioned more proximal, more cranial, and more flexed in early swing phase when compared to late swing phase at the identical femorotibial flexion angle (Fig. 6). This offset likely contributed to the lack of a statistical linear correlation with the femorotibial flexion angle. The cause of this offset upon entering and exiting swing during trotting remains to be clarified. Potential causes include the varying magnitude at which the pelvic limb musculature, particularly the quadriceps muscle group, are contracting and acting on the patella, as well as the secondary motions of the tibia such as internal-external rotation and craniocaudal tibial translation. While an increased force of quadriceps contraction did not have significant effect on patellar movement in a human cadaveric study, an in vivo MRI study demonstrated the resting patellar position in the trochlea groove could be altered by muscular contraction during either open- or closed-chain exercises [18, 19]. Surface electromyographic studies in dogs have demonstrated widely varying patterns of vastus lateralis contraction according to differing activities, which could alter patellofemoral poses during different phases of the gait cycle as observed in our study [20]. Electromyographic studies performed concurrently with radiographic kinematic analyses might improve our understanding of how muscular contraction alters patellar kinematics in dogs.

The single-plane flat-panel imaging and shape-matching methodology utilized in this study was previously validated for femorotibial kinematics in dogs [15, 21]. The precision of this methodology for this joint was determined to be within 1.28 mm and 1.58° with an intraobserver variability of less than 0.52 mm and 0.91° for translation and rotations, respectively [21]. These values reported for the femorotibial joint are unlikely to be directly applicable for the patellofemoral joint due to differences in bone geometry. Studies performed in humans using similar methodology to evaluate the patellofemoral joint kinematics reported accuracy within 0.6° for in-plane rotations and 1.5 mm for in-plane translations [22]. Analysis in this study was confined to sagittal plane translations and rotation due to the uniplanar nature of the technique and the patellofemoral joint anatomy. Although studies have not been performed to specifically validate the accuracy of our methodology for determining patellofemoral joint kinematics in dogs, a pilot series assessing repeatability of 3-D to 2-D image registration for this joint appeared to consistent with the results observed in other studies [23, 24].

The 3-D patella model was approximated to remain central within the trochlear groove such that the center of the patella’s articulating surface remained as congruent as possible with the trochlear groove in the axial plane. We utilized the 3-D geometry of the trochlear groove to define medial-lateral translation as well as patellar tilt. The kinematic parameters in the sagittal plane may have been different if the assumptions were not made. Studies in humans have shown the axial plane topography of the trochlear groove could be used to predict axial plane patellar kinematics, supporting our methodology [25]. However, coronal plane rotation, conventionally known as patellar rotation, was not readily predictable by trochlear anatomy in humans [25]. Bi-plane radiographic analysis would be required to gain a more thorough intricate understanding of canine patellofemoral joint motion in vivo.

Our data should be useful as a baseline of normal patellofemoral kinematics in dogs, against which comparisons can be made in future studies. The main goal of this study was to characterize normal patellofemoral motion in order to define the change in kinematics caused by cranial cruciate ligament rupture in a future study by our group. Because the most profound femorotibial kinematic abnormalities with cranial cruciate ligament rupture occur in the sagittal plane, it is logical to expect that the patellofemoral kinematics we reported, including patellar flexion, proximodistal translation, and craniocaudal translation, may be disrupted by the condition. Indeed, cadaveric studies have found the cranial cruciate ligament rupture can alter patella alignment and patellofemoral contact mechanics [4]. Patellofemoral mechanics are also of particular interest with the surgical treatment of cranial cruciate ligament rupture as stifle extensor mechanism abnormalities frequently occur following various procedures used to address the disease [26]. Our results are likely less applicable to patellar luxation, where the major abnormalities in motion are in the coronal plane.

## Conclusions

The study used CT based models and single-plane flat-panel imaging to quantify patellofemoral motion during commonly performed daily activity in dogs. There was significant linear correlation between femorotibial flexion angle and each of the three degrees of freedom in the sagittal plane during sit and walk. The patella utilizes the full length of the trochlear groove during daily activity. Trotting induced offset, where patellar poses differed according to the phase of the gait cycle, despite identical femorotibial flexion angles. By defining normal in vivo kinematic patterns non-invasively, new knowledge of the normal canine stifle has been generated which may lead to the improvement and development of new surgical correction methods for stifle abnormalities.

## Abbreviations

CT: Computed tomography
DICOM: Digital imaging and communication in medicine

## Footnotes

1 Toshiba Aquilon 8, Toshiba American Medical Systems Inc, Tustin, CA.

2 Toshiba Infinix-I flat panel C-arm fluoroscope, Toshiba American Medical Systems Inc, Tustin, CA.

3 ITK-SNAP, Paul A. Yushkevich et al., http://www.itksnap.org.

4 Geomagic Studio, Geomagic Inc., Research Triangle Park, NC.

5 JointTrack, University of Florida, FL, http://sourceforge.net/projects/jointtrack/.

6 Matlab, MathWorks, Natick, MA.

7
^g^ SigmaPlot 12, Systat Software, Inc. San Jose, CA.
